# Wavelet Scattering-Based Classification of Subthalamic Nucleus and Substantia Nigra Microelectrode Recordings During Deep Brain Stimulation Surgery

**DOI:** 10.3390/s26216787

**Published:** 2026-10-27

**Authors:** Dorota Mularczyk

**Affiliations:** NASK National Research Institute, 01-045 Warszawa, Poland; dorota.mularczyk@nask.pl

**Keywords:** Deep Brain Stimulation, subthalamic nucleus, substantia nigra, wavelet scattering, machine learning

## Abstract

Deep Brain Stimulation (DBS) surgery offers substantial improvements in quality of life for patients with Parkinson’s disease. It consists of placing a stimulating electrode in the subthalamic nucleus (STN). Accurate and efficient localization of the target is essential because targeting errors may affect patient outcomes and the surgery is performed with the patient awake. Some systems already support neurosurgeons in the STN localization, but reliably separating the STN from the nearby substantia nigra pars reticulata (SNr) remains challenging. This study systematically evaluates the wavelet scattering transform for distinguishing STN from SNr under realistic DBS surgical constraints, where low-frequency information is unavailable and each recording must be classified independently in real time. Varying both the scattering parameters and the model complexity shows how these factors together influence performance. Classifiers ranging from support vector machines to temporal convolutional networks were capable of achieving balanced accuracy of 0.83–0.84 and receiver operating characteristic area under the curve (ROC AUC) values of 0.91–0.93. This suggests that most discriminative information is captured by the wavelet scattering representation itself, resulting in robust performance across models of different complexity. The scattering-based approach achieved consistently higher performance than the evaluated wavelet packet-based baseline and highlights the potential of scattering-based representations for reliable STN localization during DBS surgery. However, the findings were obtained in a single-author, non-preregistered study using data acquired at a single center with a single recording system. Independent multicenter validation will therefore be necessary before broader clinical application can be considered.

## 1. Introduction

Deep Brain Stimulation (DBS) is a neurosurgical procedure which, as the name suggests, consists of modulating neural activity by electrical stimulation [1]. It is a common treatment method for movement disorders, such as Parkinson’s disease (PD), tremor, and dystonia [2]. It can also be used for pain syndromes [3], epilepsy [4], and some psychiatric disorders [5]. DBS has been approved for obsessive–compulsive disorder, and for many others it is currently under investigation.

DBS offers a substantial increase in the quality of life even for patients whose condition resists other methods of treatment. However, it requires a high level of expertise and precision from the neurosurgeon. It consists of placing a stimulating electrode (or a set of them) in a specific brain region and leaving it there permanently. The brain region where the electrode should be placed depends on the disease being treated, but typically it is small and located deep inside the brain. What makes the surgery even more challenging is the fact that the target region can be very similar morphologically to the nearby regions. As a consequence, its exact location often cannot be determined with standard imaging methods like computed tomography or magnetic resonance imaging (MRI).

In such cases, the localization is performed intraoperatively, based on the electrical signals registered inside the brain by a set of microelectrodes [6]. These electrodes, different from the stimulating ones, are advanced through the brain step by step. At each point, they register spikes of voltage produced by the neuron cells near the electrode, mixed with the noise from the neurons in the broader surroundings. The characteristics of the signal allow an experienced neurosurgeon to determine in which brain region the signal was recorded. It is important to find the location of the target region quickly and precisely. Time matters for the comfort of the patient, who remains awake, since the surgery is performed with only local anesthesia. Thanks to this, some adverse effects caused by wrong electrode placement can be noticed and corrected during the surgery.

PD is one of the diseases commonly treated with DBS. The treatment procedure in this case involves all the challenges described above. The subthalamic nucleus (STN), which is the target region, is small (only around 3 × 5 × 12 mm [7]). Although MRI has been proven useful in localizing it, some discrepancies exist. They arise from the fact that MRI visualizes an iron-dense structure that overlaps with the STN, not the STN itself. Also, the visualization is performed preoperatively. During the surgery, the anatomic structures of the brain may shift due to cerebrospinal fluid loss and subdural air invasion. For these reasons, determining STN location intraoperatively with the use of microelectrode recordings (MERs) is still a common practice [8].

**Figure 1:**
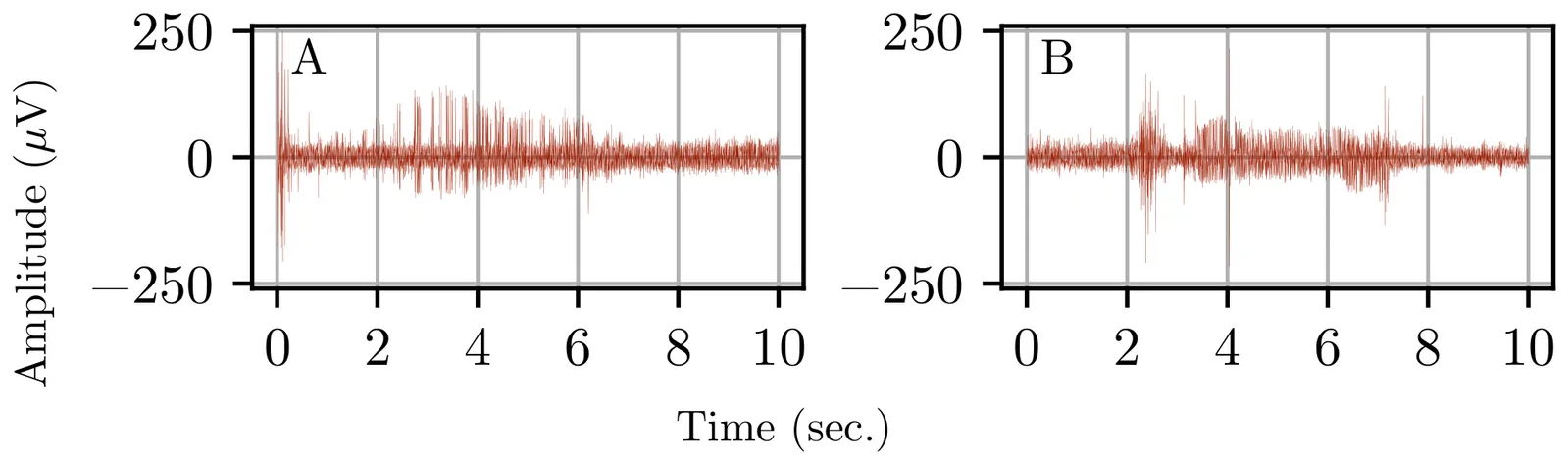
Sample recordings registered during DBS surgery in STN (**A**) and SNr (**B**).

Incorrect electrode placement can have a detrimental effect on the patient’s health [9,10] since the nearby regions perform critical brain functions [11]. It is especially difficult to distinguish between STN and the structures located ventrally to it. One such structure is the substantia nigra pars reticulata (SNr). Its characteristics are similar to those of STN. Typically, the spikes of activity appear more frequently in SNr than in STN, and the background noise is lower in the area between the two regions, but the differences are not consistent enough to provide a reliable basis for discrimination [12,13]. Sample recordings from STN and SNr are presented in Figure 1. Moreover, the exact topography of a brain is highly patient-dependent. In some patients, SNr is separated from STN by a few millimeters, while in others the distance is not noticeable. Failure to detect the SNr poses a serious risk: ventral to it lies the optic tract, and damage to this structure may result in permanent visual impairment.

The potential of machine learning to support neurosurgeons in DBS surgeries has been noticed and investigated for years [14]. Correct classification of brain regions based on MERs can facilitate electrode placement. Numerous studies use traditional machine learning approaches, such as hidden Markov models [9,15], support vector machines [9], and k-nearest neighbors (KNN) [16], among others, but neural networks have also been applied in the field [17,18]. Features used for classification are often handcrafted, spike-dependent (such as firing rate or inter-spike interval), or independent (for example, variance or skewness calculated over a signal segment of fixed duration). Several reviews summarizing advances in machine learning use for DBS in PD are available [19,20].

While existing systems achieve clinical-grade results for STN detection [18,21,22], distinguishing between STN and SNr remains a challenge. The hidden Markov model was shown to achieve good results when trajectory history information was included in addition to MERs [9]. Chaovalitwongse et al. [23] undertook the classification of STN, SNr, and another nearby structure (zona incerta) based on handcrafted features and on frequency features with KNN, Bayesian, and tree classifiers. Schiaffino et al. [16] used KNN to classify STN and SNr, and their results confirm that SNr is more difficult to classify in a DBS-realistic setup.

Better distinction between STN and SNr can be achieved using Local Field Potentials or by analyzing signal activity at frequencies below 50 Hz [9]. Koirala et al. [24] analyzed spiking rates and background activity for localization of both STN and SNr and pinpointed increased beta-activity in STN as a feature that can help distinguish it from SNr. However, beta-activity is generally unavailable or strongly attenuated from conventional MER registered during DBS surgery, since these recordings are usually made using high-impedance electrodes, and low-frequency components are, as a rule, filtered out by the hardware during signal acquisition.

The application of machine learning techniques in DBS has also been investigated in contexts other than target localization, for example, to predict treatment outcomes and postoperative complications [20,25,26,27,28]. Explainable machine learning has been employed to explain the variability in DBS outcomes in different patients [29]. The findings show that individual neurophysiology plays a major role, but also emphasize the importance of precise electrode placement. Machine learning can also facilitate the optimization of stimulation, for example, finding the stimulation frequency [30]. Recently, a significant amount of work has been put into developing so-called adaptive DBS, which allows for real-time stimulation adjustments [31,32,33].

The approach used in this work employs the wavelet scattering transform for feature extraction [34]. Wavelets are oscillations of limited duration and mean amplitude equal to zero. Convolution of a signal with dilated wavelets in a so-called wavelet transform yields coefficients that characterize the signal in a way that is resistant to deformation. Unlike the Fourier transform, this resistance is conserved even at high frequencies. The wavelet scattering transform extends the wavelet transform by the application of complex modulus and a low-pass filter, which introduces non-linearity and produces translation-invariant representations.

Despite the growing use of wavelet scattering in biosignal analysis, its application to STN versus SNr discrimination using conventional intraoperative MER recordings has not been systematically investigated.

The main contributions of this study can be summarized as follows: (1) a demonstration that the wavelet scattering transform provides robust and effective feature representations for STN/SNr discrimination; (2) a systematic evaluation of how different scattering parameters and model complexities affect classification performance; (3) the development of a model that achieves reliable STN vs. SNr classification under clinically realistic intraoperative conditions (24 kHz sampling, no low-frequency information) and supports single-recording, real-time classification suitable for DBS surgery.

## 2. Materials and Methods

### 2.1. Wavelet Scattering Transform

Finding a concise yet informative signal representation is especially important when the amount of data available for model training is limited. The wavelet scattering transform is known to be a suitable candidate for this task. The application of the wavelet scattering transform to a signal starts with the construction of a filter bank. Each filter used in this study is a Morlet wavelet obtained from a mother wavelet ψ(t) through dilation:

(1) $$\psi_{j,q}\left(t\right)=2^{-(j+q/Q)}\psi \left(2^{-(j+q/Q)}t\right),$$

where ψ(t) is the mother wavelet, *j* denotes the octave index, and q=0,…,Q−1 identifies a wavelet within a given octave. The parameter *Q* determines the number of wavelets per octave, while the maximum scale is determined by the parameter *J*. The zeroth-order scattering coefficient is obtained by averaging the signal at the coarse scale $2^{J}$ (equivalent to applying a low-pass filter at that scale), yielding a smoothed representation:

(2) $$S_{0}x\left(t\right)=x*\phi_{2^{J}}\left(t\right),$$

where $\phi_{2^{J}}$ denotes a low-pass filter operating at scale $2^{J}$. Then the original signal is convolved with the filters from the bank. In the next stage, a complex modulus is applied to the output of the convolution. The resulting first-order scattering coefficients are given by:

(3) $$S_{1}x\left(t\right)=\left|x*\psi_{j_{1},q_{1}}\right|*\phi_{2^{J}}\left(t\right).$$

The output of the modulus follows two separate paths: the first is the application of a low-pass filter in order to obtain the first-order coefficients; the other path is passing the modulus output to another convolutional layer, which exploits the filter bank again. Then, a complex modulus is applied again, followed by a low-pass filter to obtain the second-order coefficients:

(4) $$S_{2}x\left(t\right)=|\left|x*\psi_{j_{1},q_{1}}\right|*\psi_{j_{2},q_{2}}|*\phi_{2^{J}}\left(t\right).$$

The final output of the wavelet scattering transform used in this study is a concatenation of the 0th, 1st, and 2nd order coefficients.

### 2.2. Dataset

The experiments were conducted using the dataset accompanying the paper [18], released under a CC BY-NC 3.0 license. It contains 1440 MERs originating from the STN and 94 from the SNr, from 46 patients. All signals were acquired at a sampling rate of 24 kHz, with durations ranging from 5.2 to 10 s. The recordings were collected using a Medtronic LeadPoint system during DBS surgeries.

### 2.3. Data Preprocessing

Each recording was cropped to a uniform length of 5 s by retaining the initial 5-s segment and transformed using a 1D wavelet scattering provided by the Kymatio Python library (version 0.3.0) with a PyTorch frontend [35]. The transform was computed for four values of the scale parameter J (2, 6, 8, and 12), with the number of wavelets per octave set to the same value (Q = J).

In accordance with Kymatio’s recommended preprocessing, zeroth-order scattering coefficients were discarded, and the remaining coefficients were log-transformed (after adding $10^{-9}$ for numerical stability). For each setting of J and Q, two versions of the feature vectors were generated: (1) the time-resolved scattering features obtained directly from the transform after preprocessing, and (2) time-invariant representations obtained by averaging each preprocessed coefficient over the temporal dimension.

For the time-invariant representation, each preprocessed scattering coefficient was averaged over its temporal dimension:

(5) $${\bar{S}}_{k}=\frac{1}{T_{k}}\sum_{t=1}^{T_{k}}S_{k}\left(t\right),$$

where $S_{k}\left(t\right)$ denotes the *k*-th preprocessed scattering coefficient and $T_{k}$ is its temporal length.

The resulting feature dimensions were: (5, 30,000) for J/Q = 2/2, (95, 1875) for J/Q = 6/6, (233, 469) for J/Q = 8/8, and (800, 29) for J/Q = 12/12. The first dimension corresponds to the number of scattering coefficients and the second to their temporal resolution.

### 2.4. Models

**Figure 2:**
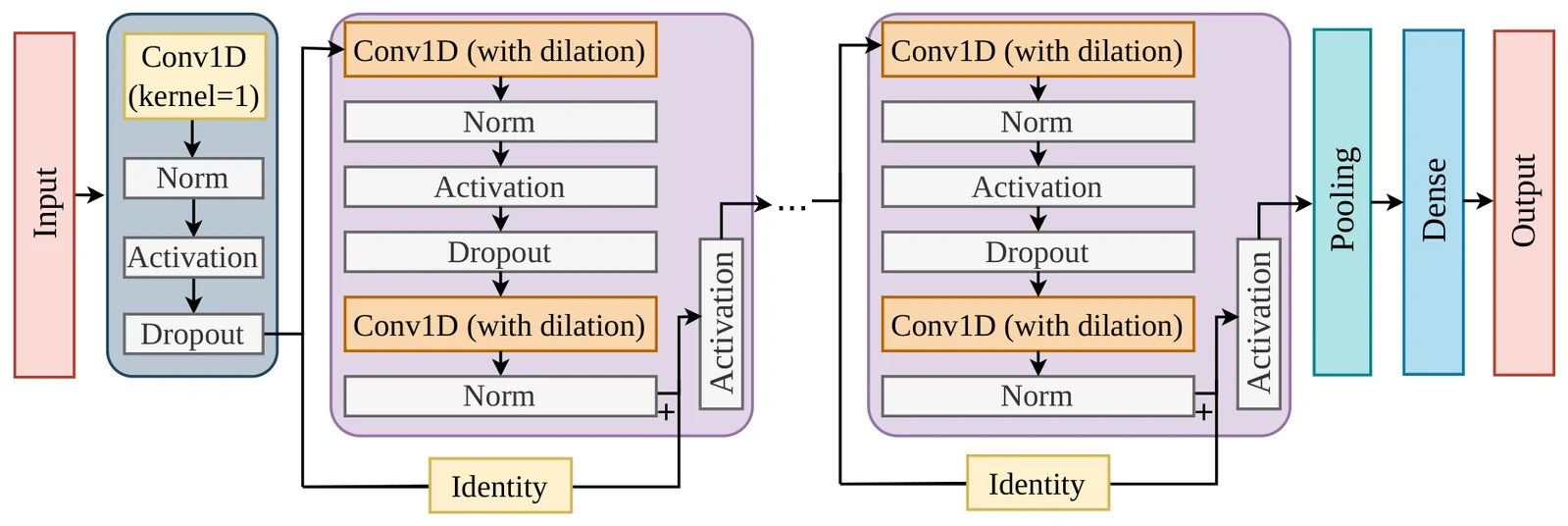
A generic architecture of a Temporal Convolutional Network.

Three classes of models were trained and compared in order to check what level of complexity is needed to obtain satisfactory results with features extracted through the wavelet scattering transform. The Support Vector Machine (SVM) model was chosen as the simplest baseline, and a multilayer perceptron (MLP) was used as a more powerful alternative. Both models used features averaged along the temporal dimension. In order to check if the temporal dimension contains information that could improve performance, the third model class was included: the Temporal Convolutional Network (TCN) [36], whose dilated convolutions and residual skip connections allow the model to learn temporal relationships across the scattering coefficient sequences. A scheme of a TCN network is presented in Figure 2.

#### 2.4.1. Support Vector Machine

SVM models with the radial basis function (RBF) kernel were trained and validated with the use of the scikit-learn Python library [37] on the preprocessed scattering coefficients averaged along the time dimension. Different values of the regularization parameter C (0.1–100) and several settings of the gamma parameter (“scale”, “auto”, 0.01, 0.001, 0.0001) were tested. For each J and Q setting, the best model was selected based on the receiver operating characteristic area under the curve (ROC AUC).

#### 2.4.2. Multilayer Perceptron

Neural networks consisting of 1–3 dense layers with dropout, normalization layers, and ReLU activation were implemented in PyTorch [38] (version 2.6.0) and trained on the preprocessed scattering coefficients averaged along the time dimension. Training was stopped after 10 epochs without loss improvement. The hyperparameters were optimized using Optuna [39] with the Asynchronous Successive Halving Algorithm (ASHA) pruner over 500 trials, and the selection was based on the ROC AUC. Five combinations of layer sizes and numbers of layers were tested for each feature set. Network size depended on the dimensionality of the input vectors. For the shortest 5-element vectors (J/Q = 2/2), 1- and 2-layer networks were used, with layer sizes between 2 and 16. For the longest 800-element vectors (J/Q = 12/12), the networks consisted of 2 or 3 layers with sizes ranging from 32 to 512. Dropout ranged from 0 to 0.5, learning rate from 0.0001 to 0.01, and weight decay from 0 to 0.01. Both batch and layer normalization were tested, with batch sizes of 64 or 128.

#### 2.4.3. Temporal Convolutional Network

TCNs were implemented in PyTorch and trained on feature vectors without averaging along the time dimension. ReLU activation was used, and the batch size was 32 for J/Q = 2/2 features and 64 for all other settings. Hyperparameter optimization was performed in Optuna with 200 trials and the ASHA pruner. Training stopped if the validation loss did not improve for 6 epochs. The best hyperparameters were selected based on the ROC AUC. Dropout ranged from 0 to 0.5, weight decay from 0 to 0.01, learning rate from 0.0001 to 0.003, and either batch or layer normalization was used. Due to significant differences in feature dimensionality, the search ranges for some hyperparameters depended on J and Q. For J/Q = 2/2, the number of residual blocks varied between 4 and 6 with a kernel size of 3. For the remaining settings, the number of blocks varied between 3 and 5, and the kernel size was 3 or 5. The searched ranges for hidden channels were 3–8 for J/Q = 2/2, 32–128 for J/Q = 6/6, and 64–192 for the remaining cases. Different exponential and nearly flat dilation schedules were generated and adjusted to achieve a receptive field of approximately T/8, where T is the temporal dimension (ranging from 29 for J/Q = 12/12 to 30,000 for J/Q = 2/2).

### 2.5. Model Evaluation

To obtain comparable performance estimates, a nested k-fold cross-validation with 5 outer folds and 3 inner folds was performed for all models included in the study. All recordings from a given patient were assigned to the same fold at both levels of the fold hierarchy (outer and inner) to prevent data leakage. In each of the outer folds, there were 279–300 STN recordings and 12–24 SNr recordings (that is, SNr constituted 4–8% of each outer fold). There were 8–10 patients per outer fold. Not all of them had their SNr signals recorded, but at least 5 per fold did.

After each split (inner and outer), the data were standardized using the mean and standard deviation computed on the training set.

The hyperparameters were tuned separately for each of the outer training folds using the three inner folds. The decision threshold for each outer fold was determined based on the out-of-fold predictions of its inner folds to maximize the balanced accuracy. Then, final models were trained on the outer training folds. In the case of MLP and TCN, the number of training epochs was set to the ceiling of the mean of the best-epoch values from the inner-fold trainings. These models were used for inference on the outer test folds, using the determined decision threshold. The outer test had no influence on the choice of hyperparameters, the decision threshold, or the number of training epochs.

During training, the imbalance was addressed by using class weighting for the SVM models and a weighted random sampler for the neural networks. No balancing was applied during the evaluation, as the selected metrics provide reliable performance estimates even under class imbalance. The reported metrics are: ROC AUC, sensitivity, specificity, and balanced accuracy (defined as the average of sensitivity and specificity). The area under the precision-recall curve is not reported despite being a common metric for imbalanced datasets because it strongly depends on the exact proportion of classes in the dataset. In the dataset used in this study, the proportion of STN and SNr classes arises from the data collection protocol and does not reflect the true prevalence of STN segments, so precision-recall AUC is not an appropriate metric. For neural networks, a balanced validation loss was tracked, computed as the unweighted mean of the per-class mean losses, giving equal weight to each class regardless of its prevalence. This value was also used as the early-stopping criterion during training.

To assess whether the observed differences between models could be attributed to random variation, a bootstrap analysis was performed on pooled out-of-fold predictions. Balanced accuracy differences between selected model pairs were estimated using 10,000 bootstrap resamples. Two-sided *p*-values and 95% confidence intervals were then calculated from the resulting bootstrap distributions.

### 2.6. Reference Baseline

In order to demonstrate the effectiveness of wavelet scattering transform in feature extraction for brain region classification, an alternative method, based on [40], was implemented. Instead of the wavelet scattering transform, the wavelet packets transform was used as implemented in the pywavelets Python library [41]. The Daubechies 4 wavelet was used in symmetric mode. The decomposition was performed at 4 levels. Four features were extracted from each set of computed coefficients: mean, variance, skewness, and kurtosis. The SVM model was trained using the same procedure as for the wavelet scattering features (nested k-fold cross-validation, testing various values of C and gamma hyperparameters). Additionally, a random forest classifier was trained, since in the source article it outperformed SVM. The tested hyperparameters included 50–1000 estimators, maximum depth either null or from the 3–20 range, and at least 1–10 samples per leaf. The imbalance was addressed by class weighting.

### 2.7. Statistical Analysis of Scattering Coefficients

Time-averaged scattering coefficients obtained with J/Q = 8/8 were analyzed statistically to compare STN and SNr recordings. Normality of the coefficient distributions was evaluated using the Shapiro-Wilk test, and although some deviations from normality were detected, mean and median coefficient values were generally similar, indicating that these deviations were moderate. Welch’s *t*-test was selected a priori to assess the differences in coefficient values between STN and SNr recordings because it does not require the assumption of equal variances and is known to be robust to moderate departures from normality. Statistical significance was determined at the α = 0.05 level. To account for multiple comparisons across the 233 coefficients, the Benjamini–Hochberg procedure was applied to control the false discovery rate at 0.05. As a non-parametric sensitivity analysis, the Mann–Whitney U test was also performed using α = 0.05.

### 2.8. Latency Estimation

Latency of the signal classification was estimated for: (1) extracting scattering features; (2) forward pass of the MLP and TCN models; (3) end-to-end procedure, including cropping the signal to the desired window of 5 s, transferring it to the chosen device, extracting scattering features, and forward pass of the model. Results are reported only for MLP and TCN models. The latency of SVM inference was not measured separately because the model operates on the same time-averaged feature vectors as the MLP and its computational cost is negligible compared with the feature-extraction stage. All measurements were performed with a batch size of one, i.e., a single 5-s window processed at a time. Each measurement consisted of 5 warm-up passes and 100 timed passes, which were averaged. Synthetic random signals were used for latency benchmarking and the models with the most demanding architectures were initialized with random weights, since the latency is independent of both the exact data values and the weights. For simplicity, the same model architecture was used for each J/Q setting. The estimation was performed on a laptop-class CPU (11th-gen Intel Core i5-11400H, 6 cores, 12 threads, 32 GB RAM) and an NVIDIA GeForce RTX 3050 Laptop GPU, 4 GB VRAM.

## 3. Results

**Figure 3:**
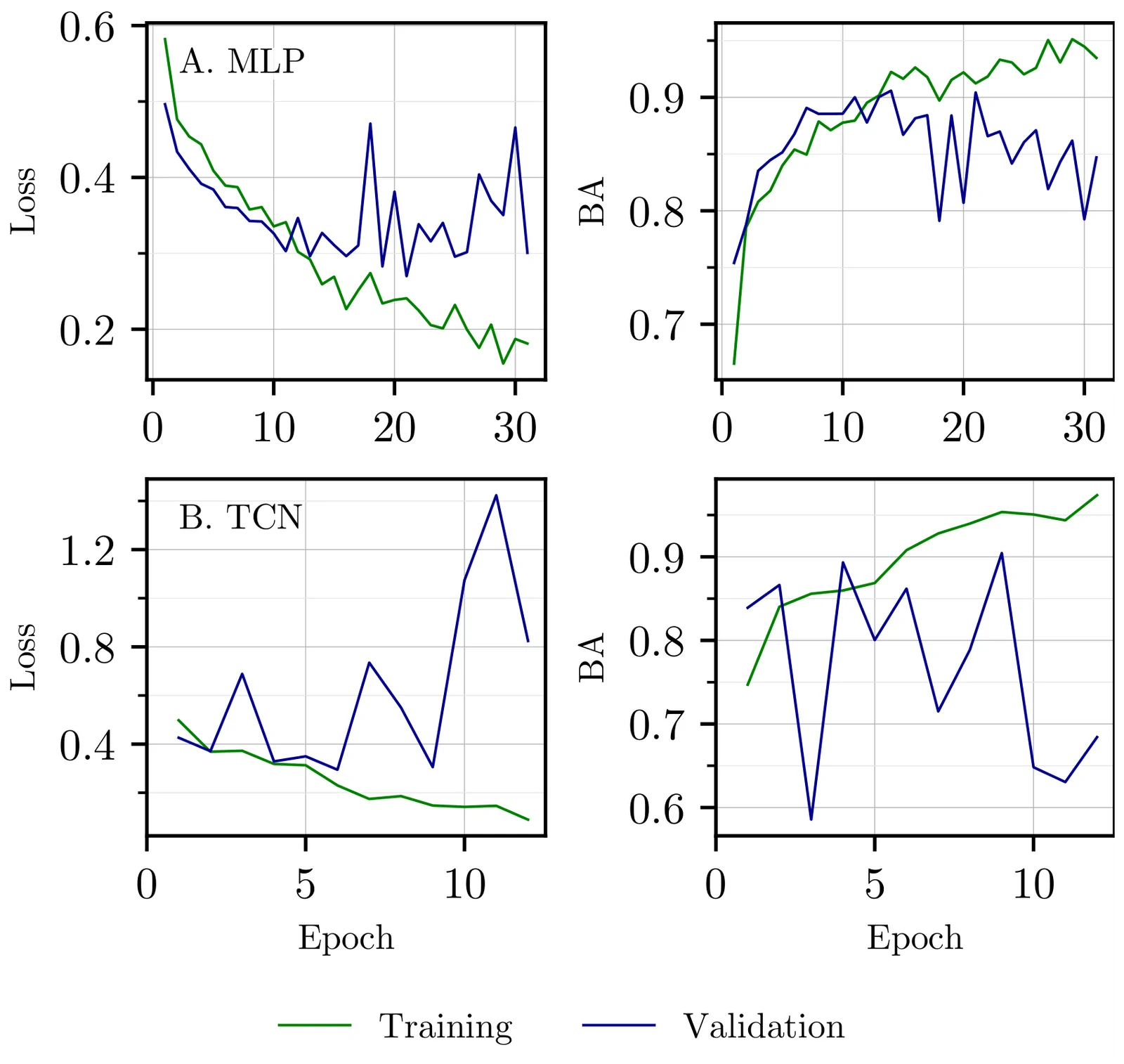
Training and validation curves for MLP ((**A**), upper) and TCN ((**B**), lower) models from a representative inner cross-validation fold. Balanced loss is shown in the left plots, and balanced accuracy (BA) in the right plots.

Figure 3 presents training and validation curves for representative MLP and TCN models. TCN requires fewer training epochs to converge, but in both cases a noticeable degree of overfitting is visible.

Metric values obtained from cross-validation for the best models after threshold tuning are presented in Table 1. All values are at least 0.75, and balanced accuracy ranges from 0.80 to 0.84. Due to the small number of SNr samples, the standard deviations of the cross-validation metrics are relatively high (0.06–0.13 for balanced accuracy, which is greater than the typical differences observed between the models). Although the differences between the classifier types were small, one trend that remained consistent across experiments was the strong performance of feature sets obtained with J/Q = 8/8, which were among the best-performing configurations for all model classes. However, bootstrap comparisons between the best- and worst-performing J/Q configurations within each model family did not reveal statistically significant differences in balanced accuracy (all 95% confidence intervals included zero). Likewise, no statistically significant differences were observed between the best-performing SVM, MLP, and TCN models (J/Q = 8/8).

**Figure 4:**
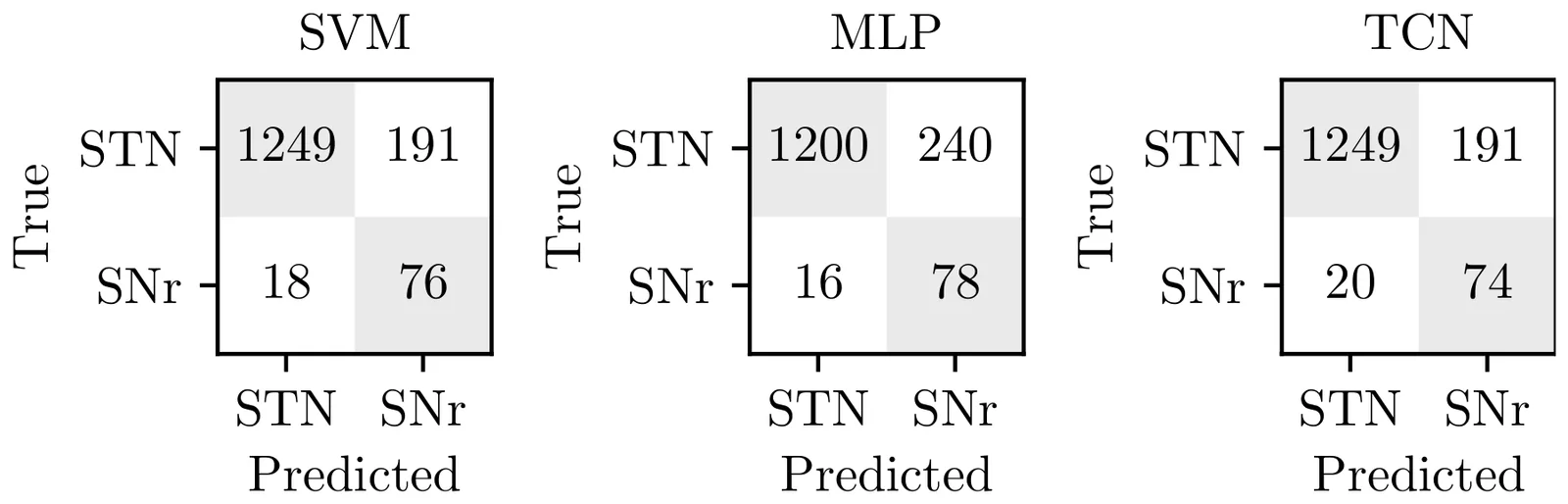
Confusion matrices for the best-performing SVM, MLP, and TCN models trained on the features based on the scattering coefficients obtained with J/Q = 8/8.

Confusion matrices for the best-performing models of each kind (J/Q = 8/8) are presented in Figure 4. For all models, the number of STN recordings misclassified as SNr is higher than the number of SNr recordings misclassified as STN. This difference is expected given the substantial class imbalance in the dataset.

### 3.1. Receiver Operating Characteristic Curve Analysis

**Figure 5:**
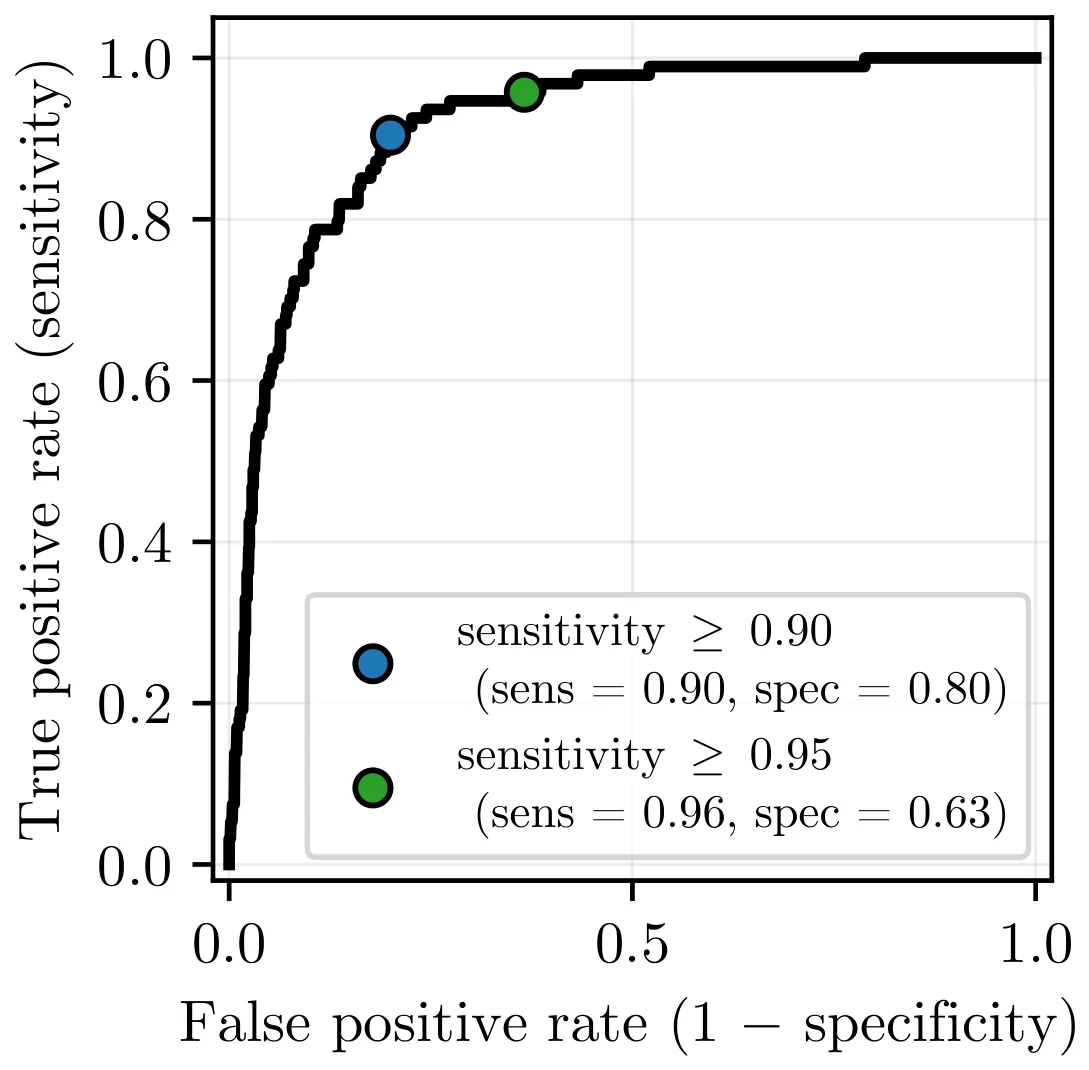
ROC curve for pooled out-of-fold predictions of the SVM model trained on scattering features extracted with J/Q = 8/8. The blue and green points indicate operating points corresponding to SNr sensitivities of at least 0.90 and 0.95, respectively.

From the clinical perspective, it is particularly important to avoid misclassifying SNr recordings as STN, as it may lead to incorrect electrode placement. The trade-off between the specificity and sensitivity of SNr detection can be visualized using a receiver operating characteristic (ROC) curve. Figure 5 depicts the ROC curve for the pooled out-of-fold predictions of the SVM model trained on the scattering features extracted with J/Q = 8/8. The two highlighted operating points correspond to high-sensitivity SNr detection. Requiring an SNr sensitivity of at least 0.9 results in a specificity of 0.8, whereas increasing the sensitivity to at least 0.95 lowers the specificity to 0.63.

### 3.2. Baseline Comparison

In comparison to the method previously proposed in the literature [40], the feature extraction exploiting the wavelet scattering transform performs better. The results are shown in Table 1. The features based on the wavelet packets transform allowed for a balanced accuracy of 0.78 ± 0.11 for the best-performing random forest model. By comparison, all three classifier families trained on wavelet scattering representations achieved substantially higher balanced accuracies, reaching 0.83–0.84. A bootstrap comparison between the SVM model trained on scattering features with J/Q = 8/8 and the best-performing wavelet packet baseline (random forest) confirmed that the observed difference in balanced accuracy was statistically significant (mean difference 0.063, 95% CI [0.023, 0.104], *p* = 0.002).

### 3.3. Scattering Feature Analysis

**Figure 6:**
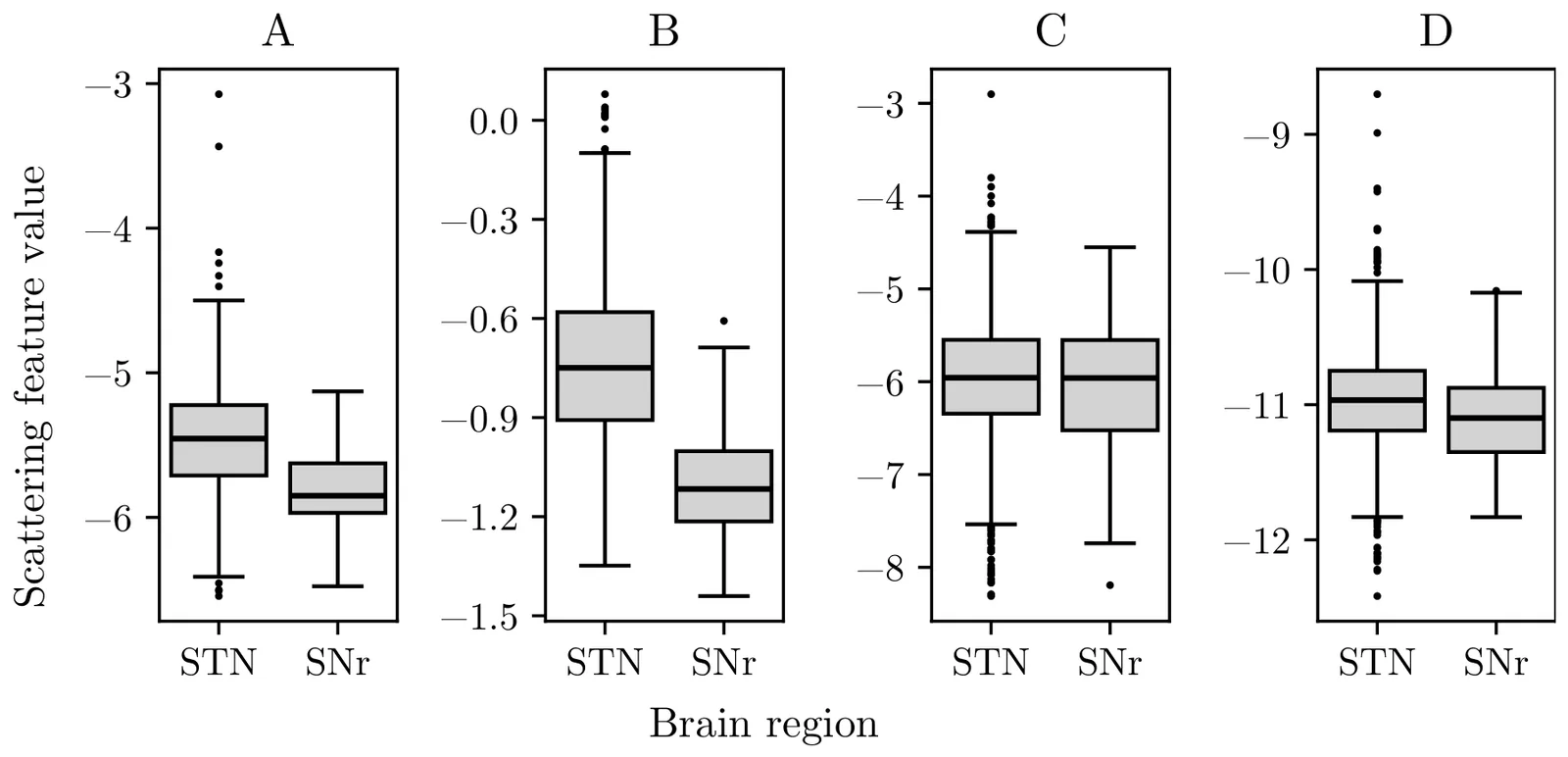
Boxplots comparing chosen feature values for STN and SNr recordings. Panels (**A**,**B**) show features with significantly different values for the two brain regions. For the feature depicted in panel (**C**), statistical tests did not show significant differences. For the feature shown in panel (**D**), the Mann–Whitney test showed no statistically significant difference, while the Welch test suggested that the difference is statistically significant. Features shown in panels (**A**–**C**) were randomly selected from the corresponding categories, whereas panel (**D**) presents the only feature exhibiting a discrepancy between the two statistical tests.

The comparison between the preprocessed and time-averaged wavelet scattering coefficients, used as features by the classifier, showed that the values derived from STN signals are typically higher (or, in fact, less negative) than those derived from SNr. The Welch *t*-test showed that the differences are statistically significant for 231 of the 233 features. Applying the Benjamini–Hochberg correction for multiple comparisons yielded the same result, with 231 features remaining significant. Similar conclusions were obtained using the Mann–Whitney U test, which identified significant differences for 230 features. Of the three coefficients identified as non-significant by the Mann–Whitney test, two were also identified as non-significant by the Welch test. Figure 6 shows the boxplots for four sample features: two for which the differences are statistically significant according to both tests, one for which the difference is not statistically significant according to either test, and one for which the difference is statistically significant according to the Welch test but not according to the Mann–Whitney test.

The constructed feature vectors contain preprocessed scattering coefficients of the first and second order. To check whether one type of coefficients is more informative and what the benefits are of using both of them, an SVM model was trained using the same procedure as before, including hyperparameter optimization, but this time once with the first-order coefficients only, and once with the second-order coefficients only. The procedure was conducted for coefficients obtained with J/Q = 6/6, J/Q = 8/8, and J/Q = 12/12. J/Q = 2/2 was omitted since it provides only the first-order coefficients. MLP and TCN models were also trained using the same procedure as before, after adjusting the sizes of the MLP hidden layers and the numbers of TCN hidden channels, which was necessary since the input tensor size is smaller when only one type of coefficients is considered. The hidden layers of the MLP model consisted of 4–48 neurons when the first-order coefficients were used and 10–168 neurons when the second-order coefficients were used. For the TCN model, the number of hidden channels was 8–48 for the first-order coefficients and 48–168 for second-order coefficients. Balanced accuracy values achieved by the models are shown in Table 2. The differences in performance between models trained on first-order, second-order, and combined scattering coefficients are small and typically do not exceed 0.02. The main exception is the MLP model, for which the representation based solely on second-order coefficients achieved a balanced accuracy of 0.85±0.09 compared to 0.80±0.05 for first-order coefficients. For this model, bootstrap analysis indicated statistically significant differences between the first-order representation and both the second-order and combined representations, whereas no statistically significant differences between coefficient types were observed for the remaining classifier families.

### 3.4. Error Analysis

The analysis of the erroneous predictions was conducted for the SVM model trained on features obtained with J/Q = 8/8 (both first- and second-order coefficients).

#### 3.4.1. Errors per Patient

**Figure 7:**
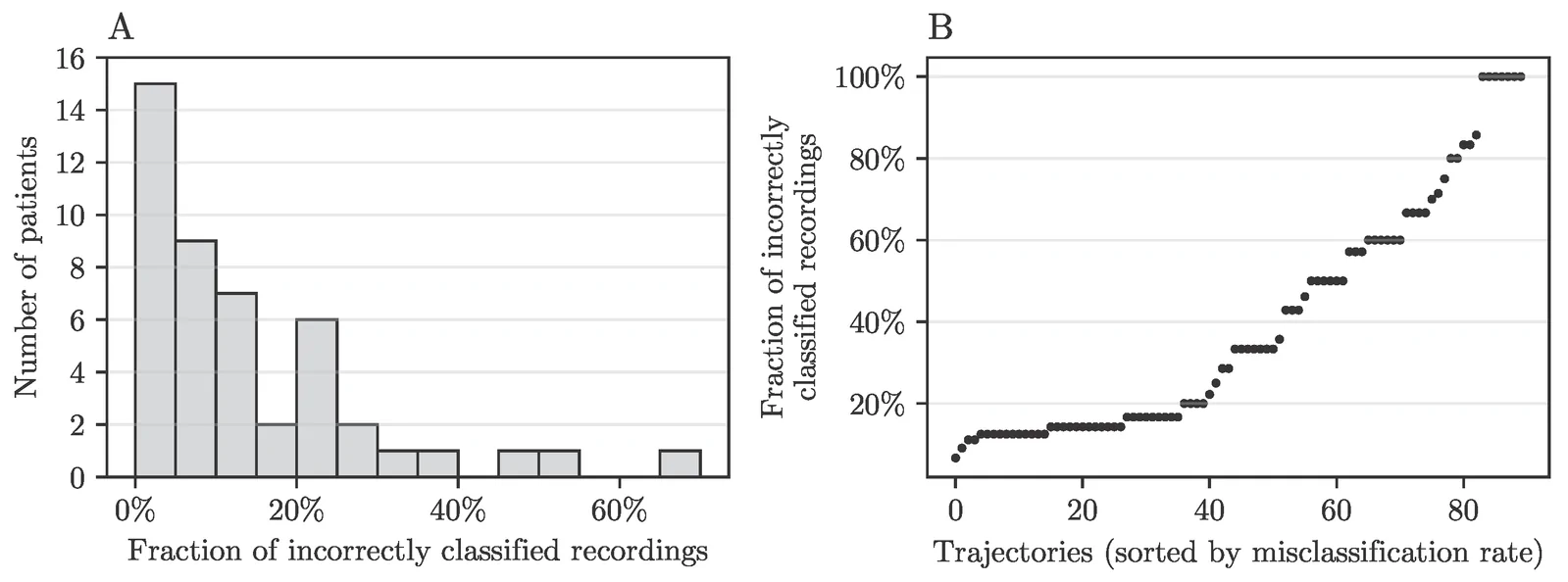
Distribution of classification errors at the patient and trajectory levels. (**A**): Histogram showing patient-level misclassification rates, defined as the fraction of recordings incorrectly classified for each patient. (**B**): Misclassification rates for individual trajectories. Each point corresponds to a single trajectory, and trajectories are sorted in ascending order of the fraction of incorrectly classified recordings. For clarity, only the 90 trajectories with a non-zero misclassification rate are shown; 166 trajectories without any misclassified recordings were omitted.

The errors committed by the model are not equally distributed among the patients. Figure 7A shows the distribution of patient-level error fractions, calculated as the proportion of incorrectly predicted recordings for each patient. The histogram depicts the number of patients whose error fraction falls within each interval. The highest error fraction is 0.68: out of 19 recordings, only 6 were classified correctly by the model. The second-highest error fraction is 0.52 and contains 10 correct predictions out of 21. Both patients did not have any signals recorded in the SNr regions, so all the errors were STN recordings classified as SNr. On the other hand, for 7 of the 46 patients, all predictions were correct.

#### 3.4.2. Errors per Trajectory

A trajectory is defined as a path along which an electrode was advanced while registering the signals. A set of recordings belongs to the same trajectory if it was registered by the same electrode in the same hemisphere and the same patient. In total, 256 trajectories were registered. In 166 of them, all recordings were classified correctly, and in 7, all recordings were classified incorrectly. Of these 256, 5 cases were not real trajectories but just single recordings (four of them were classified correctly). The maximum number of recordings per trajectory was 15, and the median was 6. Figure 7B depicts the fraction of each trajectory that was erroneously classified by the model.

#### 3.4.3. Errors per Electrode

Balanced accuracies per electrode for out-of-fold predictions were compared in order to check if the model performs worse for a specific electrode. The obtained results are presented in Table 3. Values range from 0.79 for the medial electrode up to 0.92 for the lateral electrode. While some variability is present, none of the observed values indicate a substantial degradation of performance for any particular electrode. Out-of-fold results are reported (and not the average and standard deviation from the folds) because the difference in the number of predictions from each electrode per fold would distort the results. For example, there are 0 recordings from lateral electrodes in one fold and 43 in another, or 65 recordings from medial electrodes in one fold and 112 in another.

### 3.5. Inference Latency

The times measured for feature extraction, forward pass, and the end-to-end classification procedure for MLP and TCN models with different J and Q settings are presented in Table 4. Feature extraction times were largely independent of the classifier: the only difference is that for the MLP model, the features are additionally averaged along the time dimension. The longest latencies were observed for the features extracted with J/Q = 12/12 and for the forward pass on the TCN model with the features extracted with J/Q = 2/2. Even in these cases, the end-to-end extraction and inference procedure lasted only slightly above one second. This shows that all the models included in this study meet the real-time requirements.

## 4. Discussion

Wavelet scattering transform turned out to be a robust feature extractor for STN vs. SNr classification. It performs well across a wide range of models and transform parameters. The results indicate that high classification performance can already be achieved using the lowest-dimensional scattering representation considered in this study (J/Q = 2/2), consisting of only five features. Although feature sets obtained with J/Q = 8/8 were among the best-performing configurations, all evaluated representations achieved strong results, indicating that the effectiveness of the approach is not restricted to a narrow range of transform parameters.

An important finding of this study is that TCN models did not demonstrate a consistent advantage over the simpler SVM and MLP classifiers. Since the latter operate on time-averaged scattering features, these results suggest that averaging the scattering coefficients preserves much of the information relevant for STN/SNr discrimination.

The confusion matrices reveal a larger absolute number of STN recordings misclassified as SNr than SNr recordings misclassified as STN. The reason for that is the strong class imbalance in the dataset, with STN recordings greatly outnumbering SNr recordings. This proportion does not reflect the relative clinical importance of the two types of errors. Although STN recordings are more frequent in the dataset, it is more important to avoid the classification of SNr as STN. This kind of error in real-life settings can result in the misplacement of the stimulating electrode and thus have severe consequences.

From a practical perspective, in a previously reported system [18], MERs are first acquired at multiple depths, yielding approximately 48 recordings per hemisphere, and the decision-support system is then applied to the collected recordings. Predictions are expected to be available within approximately two minutes. The measured latency suggests that the proposed approach is compatible with this type of DBS decision-support workflow. Within such a workflow, the STN/SNr classifier could serve as one component of a broader localization system, since distinguishing STN from other neighboring brain regions also remains important.

Prior studies often do not report SNr-specific metrics or use unavailable low-frequency components. Schiaffino et al. [16] use MERs for SNr classification and report sensitivity of 0.78 ± 0.065 and specificity of 0.75 ± 0.05 achieved with the fuzzy form of the KNN classifier. Chaovalitwongse et al. [23] report the sensitivity of 0.8618 and specificity of 0.8772 for a classification tree trained on handcrafted features, while the frequency-based features perform worse, with sensitivity below 0.6.

In [40], the authors use wavelet-packet features and achieve an accuracy of 0.78 and an F-score of 0.84 on the STN vs. post-STN classification task with a random forest classifier, while SVM classifiers perform worse. Yet, post-STN recordings may not originate uniquely from SNr, so the results cannot be compared directly. When applied to the present dataset, the proposed wavelet packet method performed worse than the wavelet scattering approach (balanced accuracy of 0.78 ± 0.11 for the best model based on wavelet packet features, compared to balanced accuracies ranging from 0.80 to 0.84 obtained with wavelet scattering representations). However, while different values of J and Q were systematically evaluated for the wavelet scattering transform, the parameters of the wavelet packet feature extraction were adopted from the original publication and were not optimized. This asymmetry should be taken into account when interpreting the comparison results, since it may have partly contributed to the observed advantage of wavelet scattering.

When extracting the wavelet packet features, downsampling the signal and removing unstable fragments, as the authors suggested, led to significantly worse results; therefore, the reported metrics were obtained without these operations. The advantages of the wavelet scattering transform are the stability of the signal representation, including robustness to deformations, and the fact that the scattering coefficients can be used directly as discriminative features, unlike wavelet packet approaches that often rely on handcrafted statistics such as the mean, variance, skewness, and kurtosis. The two approaches have already been compared for the classification of electromyogram [42] and electrocardiogram signals [43], and in both cases wavelet scattering performed better.

The comparison of results obtained by the models trained on feature sets based on first-order and second-order scattering coefficients did not reveal a consistent advantage of either representation. Although statistically significant differences were observed for the MLP model, similar effects were not found for the remaining classifier families. At the same time, all coefficient variants yielded strong classification performance, suggesting that discriminative information relevant for STN/SNr classification is contained in both first- and second-order coefficients. Combining both coefficient types also failed to provide additional performance gains.

## 5. Limitations

This study has several limitations. First, the number of available SNr recordings was substantially smaller than the number of STN recordings, which contributed to relatively high variability of the cross-validation estimates. Second, all experiments were conducted using a single publicly available dataset acquired in a specific clinical setting. Although patient-level cross-validation was employed to prevent data leakage and to address anatomical variability, the performance of the proposed method on data collected at other centers, using different acquisition setups and hardware, requires further investigation. The robustness to electrode impedance also remains unexamined. Consequently, independent external validation remains necessary.

Regarding the comparison with the wavelet packet approach, it must be noted that, following the original publication, the wavelet packet parameters were not optimized, as opposed to a systematic scan of J and Q parameters of wavelet scattering. Therefore, part of the observed advantage of wavelet scattering may be related to this asymmetry in parameter exploration.

Finally, since this study was conducted by a single author without independent methodological oversight or preregistration of hypotheses and analytical procedures, some degree of post hoc interpretation cannot be completely excluded.

## 6. Conclusions

The challenge addressed in this study was to develop a classifier capable of differentiating between STN and SNr structures based on DBS MER data. The potential of the wavelet scattering transform as a feature extraction method was evaluated by examining the performance of models with different complexity levels across different values of the scattering parameters J and Q.

The experiments show that the features extracted using the wavelet scattering transform support accurate classification of STN and SNr. Balanced accuracies of 0.83–0.84 were achieved across all evaluated model classes, including SVM, MLP, and TCN, with no consistent advantage of more complex architectures or preserving the temporal dimension of scattering coefficients. Although J/Q = 8/8 consistently yielded among the best results, performance differences between scattering parameter settings were generally small, and even the lowest-dimensional representation achieved strong classification performance. It was also shown that wavelet scattering features allow for better performance than previously proposed wavelet packet features.

These findings highlight the potential of scattering-based features for reliable, real-time decision support during DBS surgery. At the same time, the results should be interpreted in light of the limitations of the study, including the use of a single-center dataset and the absence of preregistration or independent methodological oversight. Future work should focus on independent multicenter validation of the proposed approach, including data acquired using different recording systems and clinical protocols. Such validation will be necessary before the method can be considered for broader clinical application.

## Figures and Tables

**Table 1:** Performance metrics for all evaluated models after hyperparameter tuning. J/Q denote the wavelet scattering transform parameters. The last two rows contain the results for the models trained on the features obtained with wavelet packet transform (WPT): SVM and Random Forest (RF).

Model	J/Q	Balanced Accuracy	ROC AUC	Sensitivity	Specificity
SVM	2/2	0.82±0.07	0.90±0.08	0.82±0.13	0.83±0.07
	6/6	0.83±0.08	0.91±0.08	0.80±0.10	0.87±0.05
	8/8	0.84±0.07	0.93±0.07	0.81±0.11	0.87±0.06
	12/12	0.83±0.07	0.91±0.07	0.88±0.10	0.78±0.10
MLP	2/2	0.84±0.07	0.91±0.08	0.84±0.11	0.83±0.07
	6/6	0.82±0.09	0.91±0.06	0.79±0.20	0.86±0.06
	8/8	0.84±0.06	0.92±0.07	0.84±0.14	0.84±0.06
	12/12	0.81±0.07	0.90±0.07	0.81±0.11	0.81±0.06
TCN	2/2	0.81±0.09	0.90±0.09	0.79±0.19	0.83±0.10
	6/6	0.82±0.08	0.91±0.07	0.75±0.16	0.89±0.04
	8/8	0.83±0.13	0.91±0.09	0.77±0.24	0.88±0.08
	12/12	0.80±0.12	0.90±0.08	0.79±0.20	0.81±0.17
SVM (WPT)	-	0.74±0.05	0.84±0.03	0.82±0.05	0.66±0.10
RF (WPT)	-	0.78±0.11	0.87±0.09	0.77±0.16	0.78±0.14

**Table 2:** Balanced accuracy (BA) values achieved by the models trained on the features based on first-order, second-order, and combined first- and second-order scattering coefficients.

Model	J/Q	First-Order BA	Second-Order BA	First- and Second-Order BA
SVM	6/6	0.81±0.08	0.81±0.07	0.83±0.08
	8/8	0.83±0.07	0.85±0.09	0.84±0.07
	12/12	0.83±0.08	0.83±0.09	0.83±0.07
MLP	8/8	0.80±0.05	0.85±0.08	0.84±0.06
TCN	8/8	0.85±0.06	0.83±0.07	0.83±0.13

**Table 3:** Balanced accuracy values for different electrodes.

Electrode	Balanced Accuracy
Anterior	0.82
Central	0.87
Lateral	0.92
Medial	0.79

**Table 4:** Latency of a single 5-s window (largest architectures, batch 1; mean over 100 repetitions after 5 warm-up iterations). Extraction and forward were timed in separate loops, so the end-to-end value may deviate from their sum.

J/Q	Extraction (ms)	Forward (ms)	End-to-End (ms)
MLP—CPU			
2/2	22.1	0.2	22.4
6/6	213.5	0.2	214.0
8/8	362.3	0.2	363.0
12/12	1051.4	0.2	1057.2
MLP—GPU			
2/2	2.7	0.4	3.3
6/6	40.3	0.4	44.4
8/8	98.1	0.5	97.9
12/12	336.7	0.6	337.2
TCN—CPU			
2/2	22.4	1046.4	1049.7
6/6	210.7	65.6	280.5
8/8	360.5	19.1	380.1
12/12	1048.4	2.7	1045.1
TCN—GPU			
2/2	3.0	31.1	33.9
6/6	39.0	3.4	42.7
8/8	92.4	1.8	95.4
12/12	326.4	1.4	324.6

## Data Availability

The data analyzed in this study are publicly available as part of the dataset accompanying the publication [18] and released under a CC BY-NC 3.0 license. No new data were generated during this study. The code used for data processing, model training, and analysis is available from the author upon reasonable request.

## Abbreviations

The following abbreviations are used in this manuscript:

ASHA	Asynchronous Successive Halving Algorithm
AUC	Area Under the Curve
BA	Balanced accuracy
DBS	Deep Brain Stimulation
KNN	K-Nearest Neighbors
MER	Microelectrode recording
MLP	Multilayer perceptron
MRI	Magnetic resonance imaging
PD	Parkinson’s Disease
RBF	Radial Basis Function
RF	Random Forest
ROC	Receiver Operating Characteristic
SNr	Substantia nigra pars reticulata
STN	Subthalamic nucleus
SVM	Support Vector Machine
TCN	Temporal Convolutional Network
WPT	Wavelet packet transform
